# Offspring birth weight, gestational age and maternal characteristics in relation to glucose status at age 53 years: evidence from a national birth cohort

**DOI:** 10.1111/j.1464-5491.2008.02427.x

**Published:** 2008-05

**Authors:** D Kuh, G D Mishra, S Black, D A Lawlor, G Davey Smith, L Okell, M Wadsworth, R Hardy

**Affiliations:** MRC National Survey of Health and Development, MRC Unit for Lifelong Health and Ageing, Royal Free and University College Medical SchoolLondon; * Department of Social Medicine, University of BristolBristol, UK

**Keywords:** gestational age, glucose intolerance, HbA_1c_, maternal birth weight, offspring birth weight

## Abstract

**Aims:**

We investigated pathways linking offspring birth weight to maternal diabetes risk in later life by taking into account a range of prospective early-life and adult maternal factors.

**Methods:**

In a national birth cohort study, we examined the relationship between offspring birth weight and maternal glycated haemoglobin (HbA_1c_) at age 53 years in 581 mothers who had a first birth between age 19 and 25 years, and had data on potential confounders or mediators.

**Results:**

Mean age at first birth was 21.5 years. After adjustment for maternal body mass index (BMI), mean percentage change in maternal HbA_1c_ per kilogram increase in offspring birth weight was −1.8%[95% confidence interval (CI) −3.5, −0.1; *P* = 0.03]. This relationship was mostly accounted for by gestational age that was inversely related to maternal HbA_1c_ (−0.9%; 95% CI −1.5, −0.4; *P* = 0.001). Other risk factors for high HbA_1c_ were smoking and high BMI at 53 years. There was a significant interaction between offspring birth weight and maternal childhood social class (*P* = 0.01). Mothers from a manual background with higher birth weight offspring had lower HbA_1c_ (BMI adjusted: −3.1%; 95% CI −5.0, −1.1); this was not observed for mothers from a non-manual background (BMI adjusted: 1.9%; 95% CI −1.3, 5.0).

**Conclusions:**

Short gestational age and low offspring birth weight may be part of a pathway linking impaired early maternal growth to diabetes risk in later life. A second possible pathway linking higher offspring birth weight to later maternal glucose status was also identified. These potential pathways require further investigation in cohorts with a wider maternal age range so that the early targeting of public health initiatives can be assessed.

Diabet. Med. 25, 530–535 (2008)

## Introduction

Reproductive function, in terms of menstrual patterns, age at first pregnancy, parity and pregnancy outcome, is not only integral to women's overall health and wellbeing from menarche to menopause, but is increasingly recognized as a sentinel of chronic disease in later life [1].

With regard to pregnancy outcomes, a series of studies has shown that mothers of smaller babies tend to live shorter lives, and are more likely to die from cardiovascular disease [2, 3]. They are also more likely to have diabetes and/or insulin resistance in later life [4–6]; these are important risk factors for cardiovascular disease. Some studies show a ‘U’-shaped association between offspring birth weight and subsequent diabetes risk.

The associations between lower offspring birth weight and increased risk of maternal diabetes and cardiovascular disease do not appear to be explained by adult maternal characteristics, such as smoking or socioeconomic disadvantage, which could both lower the birth weight of offspring and increase chronic disease risk. Nor do these associations appear to be due to shorter gestational age or other pregnancy complications that may result in a baby of lower birth weight, although in one study offspring gestational age accounted for some of the inverse relationship between offspring birth weight and maternal diabetes mortality [6]. The inverse association between offspring birth weight and maternal diabetes and cardiovascular risk could also reflect biological programming during the mother's own prenatal or early postnatal growth periods that subsequently affects both her pregnancy outcome [1] and her risk of chronic disease [7]. The upturn in risk for mothers of the heaviest babies in studies that show a ‘U’-shaped relationship between offspring birth weight and maternal diabetes [4, 6] probably reflects a group of women with gestational diabetes having heavier babies and being at greater risk of diabetes later in life [8].

We examined the relationship between glycated haemoglobin (HbA_1c_) at age 53 years and the birth weight of first-born offspring in mothers participating in the Medical Research Council (MRC) National Survey of Health and Development, a prospective UK birth cohort study. HbA_1c_ corresponds to mean plasma glucose concentrations and high HbA_1c_ affects diabetes risk in people without diabetes [9]. The benefits of this study are the prospective data on offspring gestational age, the mother's own birth weight, lifetime socioeconomic position, adult smoking habits, height, weight, and weight change since early adult life; they allow us to test possible explanations for any observed association between offspring birth weight and maternal HbA_1c_.

## Material and methods

The MRC National Survey of Health and Development is a cohort of 2547 women and 2815 men followed since their birth in March 1946, so far until age 53 years [10, 11]. The sample remains generally representative of the British population born at the same time [11]. When cohort members were aged between 19 and 25 years, a study of first-born offspring to women only was undertaken [12]. By 25 years of age, 1260 women had become mothers, of whom 1004 participated in the second-generation study. Data on birth weight recorded to the nearest quarter pound, and gestational age were extracted from the confinement records of these women. There were 996 mothers whose first pregnancy resulted in a singleton birth for whom birth weight was known. The mean birth weight for singletons was 3.32 kg for male offspring and 3.20 kg for female offspring. This study was approved by the North Thames Multicentre Research Ethics Committee and the women gave informed consent.

As part of the home visit at age 53 years, trained research nurses measured height and weight and took a non-fasting venous blood sample according to standardized protocols [13], and interviewed participants, updating previously collected information on smoking habits, live births, doctor-diagnosed diabetes, and current or most recent occupation. As described elsewhere [14], HbA_1c_ was analysed by high-performance liquid chromatography using the Tosoh A1C 2.2 Plus Analyzer (Tosoh, Tokyo, Japan). Height and weight were used to calculate body mass index (BMI). Information on current or most recent occupation was used to derive adult social class, based on the British Registrar General's standard classification, categorized as non-manual (I, II, III non-manual) and manual (III manual, IV, V). Parity was based on reported live births.

Own birth weight, recorded to the nearest quarter pound, was extracted from birth records within a few weeks of delivery in March 1946 and converted into kilograms. Childhood social class was based on information on father's occupation collected prospectively in childhood from the mother. In order to investigate if weight change since early adult life could provide a possible explanation for the association between offspring birth weight and maternal HBA_1c_, self-reported height and weight at 20 years were used to derive a measure of BMI (kg/m^2^) that was closest to the women's pre-pregnancy BMI.

Analysis was done in the 581 women with measures of HbA_1c_ at 53 years and complete data on offspring birth weight and gestational age, own birth weight, current BMI, height, smoking, parity, and child and adult social class. In this sample, diabetes was diagnosed in 16 women, with its onset occurring at a mean age of 39 years. There were no cases of childhood diabetes. In 13 cases the onset of diabetes had occurred after the first birth and in the remaining cases around the same time as the birth. The geometric mean HbA_1c_ was 5.64%, mean BMI was 27.5 kg/m^2^, 29% were smokers, 66% were from the manual social class in childhood and 35% were in the manual social class in adulthood. The 581 mothers with complete data had similar offspring birth weight and gestational age to the 415 with incomplete data. Those excluded had significantly higher mean BMI than those included. There were no differences in HbA_1c_ or any of the other possible confounding or mediating variables. Mean age at first birth was 21.5 years.

### Statistical analysis

The difference in average offspring birth weight between the sexes was accounted for by subtracting 116 g from the male offspring birth weight [4]. Offspring birth weight, first divided by quartiles and then used as a continuous measure, was examined in relation to maternal HbA_1c_ and diabetes at age 53 years and to potential confounders or mediators.

The mean HbA_1c_ for the 16 women with diabetes was 7.3%, and only six had values within the normal range (< 6.1%). As the real outcome of interest is the untreated HbA_1c_, we used a censored normal regression (STATA 8.2; StataCorp, College Station, TX, USA) to examine the relationship between offspring birth weight and maternal HbA_1c_. In this way the observed values of the diabetic women were censored, so that the actual unobserved values were assumed to be at least that value or higher. The natural logarithm of HbA_1c_ (×100) was used to reduce the right skewness of the distribution. Therefore the regression coefficients in these models can be interpreted as symmetric percentage change in HbA_1c_[15].

This model was used to adjust the relationship between offspring birth weight and maternal HbA_1c_ for maternal BMI at 53 years. Possible confounding or mediating factors, namely offspring gestational age, maternal birth weight, height, smoking, parity, and child and adult social class were then added in turn. To test whether change in BMI between 20 and 53 years of age affected the relationship between offspring birth weight and maternal HbA_1c_, we substituted BMI at age 53 with BMI change. To test whether an effect of offspring birth weight on maternal HbA_1c_ was restricted to women who had a shorter pregnancy, we included an interaction term (gestational age × birth weight) in a model with the two main effects and maternal BMI at 53 years. Similarly, we included interaction terms in models with birth weight and childhood or adult social class to test whether an effect of offspring birth weight on maternal HbA_1c_ was modified by social circumstances in childhood or adult life. This was done because evidence from other studies has suggested that associations between early growth and later cardiovascular risk are stronger for those from poorer childhood or adult circumstances than for those in better circumstances [16, 17]. All potential confounders or mediators were then entered in one final model.

## Results

Maternal HbA_1c_ at age 53 years decreased by −1.52% (95% confidence interval −3.23, 0.19; *P* = 0.08) per kg change in birth weight (Table 1). After adjusting for maternal BMI at 53 years, this relationship became stronger, with maternal HbA_1c_ decreasing by −1.80% (−3.46, −0.14) per kg change in birth weight (*P* = 0.03) (Table 2). The effect of change in BMI between 20 and 53 years of age on the relationship between offspring birth weight and maternal HbA_1c_ was similar (not shown). Gestational age was strongly associated with birth weight (Table 1) and inversely associated with maternal HbA_1c_ (−0.90%; −1.45, −0.35; *P* = 0.001) for each additional week of gestation. The percentage difference in maternal HbA_1c_ for a kg change in birth weight was considerably reduced after adjusting for gestational age (Table 2). There was no evidence that there was a stronger effect of offspring birth weight on HbA_1c_ for women with shorter pregnancies (*P*-value for the interaction term = 0.5).

**Table 1:** Characteristics (means and percentages) of women by offspring birth weight (*N* = 581)

	Offspring birth weight					
			
	< 2.92 kg (*n* = 136)	2.92–3.23 kg (*n* = 125)	3.23–3.54 kg (*n* = 170)	> 3.54 kg (*n* = 150)	Per cent difference per kg offspring birth weight (95% CI)	*P*-value
HbA_1c_ (%) (geometric mean)	5.69	5.69	5.63	5.59	−1.52 (−3.23, 0.19)	0.08
Type 2 diabetes* (%)	2.9	3.2	2.3	2.7	0.60 (0.25,1.48)†	0.3
*Potential confounders*						
Gestational age (days from expected date) (mean)	−7.1	2.6	3.0	5.4	10.8 (8.9, 12.8)	< 0.001
Maternal BMI (kg/m^2^) at age 53 years (mean)	26.9	27.5	27.6	27.9	0.6 (−0.3, 1.5)	0.2
Maternal birth weight (kg) (mean)	3.14	3.28	3.42	3.49	0.23 (0.15, 0.31)	< 0.001
Maternal height (cm) (mean)	160.1	160.0	161.7	162.3	2.14 (1.21, 3.08)	< 0.001
Smoker at age 53 years (%)	42	34	26	18	0.45 (0.31, 0.65)†	< 0.001
Childhood manual class (%)	73	66	65	60	0.57 (0.40, 0.82)†	0.006
Adult manual class (%)	47	38	32	27	0.52 (0.37, 0.74)†	0.001
Number of children (mean)	2.6	2.6	2.4	2.4	−0.14 (−0.29, 0.11)	0.07

The geometric mean for HbA_1c_ was 5.64%.

* On diabetic medication, doctor diagnosed diabetes.

† Odds ratios from logistic regression.

**Table 2:** Percent difference in HbA_1c_ at age 53 years per kilogram of offspring birth weight, unadjusted, and adjusted for potential confounders (*N* = 581)

	Percent difference per kg offspring birth weight (95% CI)	*P*-value
Unadjusted	−1.52 (−3.23, 0.19)	0.08
Adjusted for maternal BMI at age 53 years	−1.80 (−3.46, −0.14)	0.03
Additionally adjusted for:		
Offspring gestational age*	−0.95 (−2.74, 0.83)	0.30
Maternal birth weight	−1.71 (−3.41, −0.01)	0.05
Maternal height	−1.64 (−3.33, 0.05)	0.06
Smoker at age 53 years	−1.29 (−2.96, 0.37)	0.1
Childhood social class*	−1.75 (−3.42, −0.08)	0.04
Adult social class*	−1.51 (−3.19, 0.16)	0.08
Parity	−1.59 (−3.35, 0.18)	0.08

* *P*-value is 0.5 for the interaction between offspring birth weight and gestational age, *P*-value = 0.01 for the interaction between offspring birth weight and childhood social class, and *P*-value is 0.3 for the interaction between offspring birth weight and adult social class. The interactions in the models were adjusted for maternal body mass index (BMI) at age 53 years.

Maternal birth weight and maternal height increased steadily across the offspring birth weight groups (Table 1). Adjusting for these factors only slightly reduced the effect of offspring birth weight on maternal HbA_1c_ (Table 2).

Women who had lighter babies were also more likely to smoke and to come from the manual classes in childhood and adult life (Table 1). Adjusting for maternal smoking or adult social class reduced the effect of offspring birth weight on maternal HbA_1c_ (Table 2). There was evidence of an interaction between childhood social class and offspring birth weight (Table 3) such that the relationship between offspring birth weight and maternal HbA_1c_ was negative for those from a manual social class but positive for those from a non-manual social class: percent change in HbA_1c_ per kg increase in offspring birth weight for those from a manual background was −3.06% (−5.03, −1.08) and percent change for those from a non-manual background was 1.87% (−1.29, 5.04). There was no evidence of an interaction between offspring birth weight and adult social class (*P*-value for interaction = 0.3). Maternal parity was not associated with the birth weight of first-born offspring (Table 1), and adjusting for parity slightly reduced the association between offspring birth weight and maternal HbA_1c_ (Table 2).

**Table 3:** Mean HbA_1c_ for women by their offspring birth weight and by their childhood social class* (*N* = 581)

	Offspring birth weight					
			
	< 2.92 kg (*n* = 136)	2.92–3.23 kg (*n* = 125)	3.23–3.54 kg (*n* = 170)	> 3.54 kg (*n* = 150)	Percent difference per kg offspring birth weight (95% CI)	*P*-value
Childhood social class: non-manual						
HbA_1c_ (%) (geometric mean)	5.47	5.61	5.60	5.62	1.87 (−1.29, 5.04)	0.2
Childhood social class: manual						
HbA_1c_ (%) (geometric mean)	5.40	5.64	5.20	5.00	−3.06 (−5.03, −1.08)	0.002

* *P*-value for the interaction term between childhood social class and offspring birth weight was 0.01. The interaction in the model was adjusted for maternal body mass index at age 53 years.

The final model showed no effect of offspring birth weight on maternal HbA_1c_ after adjusting for all other factors (Table 4). The most important factors associated with a higher level of maternal HbA_1c_ were a higher maternal BMI (*P* < 0.001), smoking (*P* = 0.002) and shorter gestational age in first-born offspring (*P* = 0.01). The *P*-value for the interaction term between childhood social class and offspring birth weight remained significant (*P* = 0.02) when added to the final model.

**Table 4:** Percent difference (95% confidence intervals) in HbA_1c_ at age 53 years from a model containing all nine variables listed below (*N* = 581)

	Percent difference in HbA_1c_ (95% CIs)	*P*-value
Offspring birth weight (kg)	0.11 (−1.77, 1.99)	0.91
Gestational age (weeks)	−0.75 (−1.32, −0.17)	0.011
Maternal birth weight (kg)	−0.70 (−2.39, 0.99)	0.41
Maternal height (cm)	−0.06 (−0.21, 0.84)	0.41
Childhood social class (manual vs. non-manual)	−0.45 (−2.27, 1.36)	0.62
Maternal BMI at age 53 years (kg/m^2^)	0.44 (0.30, 0.60)	< 0.001
Smoking at age 53 years (yes vs. no)	3.01 (1.15, 4.87)	0.002
Adult social class (manual vs. non-manual)	1.35 (−0.49, 3.18)	0.15
Parity (number of children)	0.65 (−0.24, 1.55)	0.15

*P*-value = 0.02 for interaction term between childhood social class and offspring birth weight in final model.

## Discussion

In this cohort of mothers who had a first birth between 19 and 25 years of age, gestational age rather than low birth weight was associated with higher maternal HbA_1c_ in late middle age. Compared with previous studies that have found an inverse relationship between offspring birth weight and diabetes risk [4–6, 18], our unadjusted findings are consistent, albeit weaker. This study had the advantage of including a range of prospectively collected life course factors not always available in previous cross-sectional studies [4, 6, 18]. The most important was gestational age, which strongly reduced the association between offspring birth weight and maternal HbA_1c_. A recent Swedish record linkage study also found an inverse association between gestational age and maternal diabetes mortality and that the effect of gestational age reduced the association between offspring birth weight and maternal diabetes mortality [6]. Another study [5] has reported associations between offspring birth weight adjusted for gestational age and maternal diabetes, but did not report the associations between gestational age and diabetes risk. Other studies have found that own gestational age is inversely related with adult insulin resistance and diabetes [19, 20], cerebrovascular disease mortality [21] and risk of stroke [21, 22]. In particular, reduced risk of occlusive stroke was found for those born ≥ 36 weeks of gestation [21]. It is known that higher maternal glucose concentrations reduce length of gestation [23]. This could be due to both increased fetal growth due to changes in the intra-uterine environment and increased risk of pregnancy complications, such as maternal hyperglycaemia and pre-eclampsia [24], that lead to decisions to induce labour or advance spontaneous delivery [23].

Unlike previous studies, we were able to investigate the role of change in BMI during adult life. We found no evidence for the suggestion [25] that faster increase in adiposity in mothers who gave birth to lighter babies explains the association between offspring birth weight and maternal HbA_1c_ at age 53 years.

In women from a non-manual childhood social class, those who had higher birth weight offspring had higher HbA_1c_. This interaction needs to be interpreted with caution and may be a chance finding and thus requires replication in other studies. However, it may be that the risks associated with gestational diabetes/glucose intolerance during pregnancy (resulting in a bigger baby at birth) and diabetes/glucose intolerance in later life is more important for women from a non-manual social class than for those from a manual social class, whereas the factors linking lighter babies with increased maternal glucose intolerance may predominate in women from a manual social class [23, 26, 27].

This study was limited to mothers who had a first birth between 19 and 25 years of age. Women who had their first born before age 25 years had higher parity, and this may have increased their diabetes risk compared with women whose first child was born later. This could limit the generalizability of our findings. In our recent research [28] we found that increasing parity was associated with higher risk of diabetes, but not with higher HbA_1c_. Within this group of young mothers, adjusting for parity had little effect on the relationship between offspring birth weight and maternal HbA_1c_. Another limitation of this study is the lack of data on father's offspring, which would have provided an opportunity to unravel differences between the paternal and maternal influences on offspring birth weight and diabetes risk.

In conclusion, this study has used prospective data to investigate the link between offspring birth weight and maternal HbA_1c_ in late middle age, taking into account a broad range of maternal factors across the life course. The results show that gestational age rather than low birth weight was associated with higher maternal HbA_1c_, and this may be part of a pathway linking impaired early maternal growth to diabetes risk in later life. A second possible pathway linking higher offspring birth weight to later diabetes risk in women from a non-manual social class was also identified. The relative importance of these two pathways needs to be investigated in older and younger birth cohorts and in samples with a wider maternal age range. Research should also focus on the underlying genetic and environmental mechanisms that determine the pathways and assess the potential role for early targeting of public health initiatives.

## Abbreviations

BMI: body mass index
HbA_1c_: glycated haemoglobin
MRC: Medical Research Council
